# Notes and News

**Published:** 1888-01-07

**Authors:** 


### NOTES AND NEWS.

COLLECTED AND COMMUNICATED.

WE are requested to state that Miss Lückes has not been appointed a member of the Joint Committee of The Hospitals Association.

A HOSPITAL built on the block system, and fitted with every appliance that medical and architectural skill can suggest, has just been opened at Fairford, Wilts.

THE report of the Ayr Fever Hospital and Infirmary shows that the total number of cases treated there during the past twelve months was 529. The tables of mortality show a very gratifying result. In 54 cases of fever only two deaths occurred. In general medical cases the mortality was 1 in 14·7, while in the surgical department the average was 1 in 120.

A SUCCESSFUL ball in aid of the Blackburn Infirmary took place in the Assembly Rooms, Blackburn, on the 21st ult., about two hundred persons being present.

OWING to a bad epidemic of whooping-cough the resources of the Redland Dispensary are tried to the utmost, and unfortunately the funds of the charity are now completely exhausted. Mr. Thomas Webster has written to the *Western Daily Press*, asking for immediate subscriptions for this useful institution.

A VERY successful concert, both from an artistic and a financial point of view, in aid of the Cork Hospital for Women and Children, took place on the 20th ult., in the ball room of the Imperial Hotel, Cork. The prices charged for the seats were high, but they were well filled; those who occupied them doubtless thinking of the good object to which the money was to be devoted.

IT is proposed to erect a hospital for women at Redruth, Cornwall, and a premium of ten guineas has been offered for the best plan for the building. The committee required the assurance of subscriptions to the amount of £800 before they would proceed with the work, and it is satisfactory to know that they have received, or been promised, a hundred pounds more than this amount.

THE Nottingham Town Council have made application to the Lords Commissioners of her Majesty's Treasury for leave to sell or lease some lands belonging to them, and to build a hospital for the reception of infectious diseases with the money so obtained. A Government inquiry was held, the ssue of which was to consent to the desire of the Council The Town Clerk wisely argued that it was better to use the money obtained from the sale of the land to defray the cost of building the hospital than to invest it at 3 per cent., and then borrow the required amount at 3½ per cent.

IT is greatly to be regretted that the Hull Hospital Sunday Fund is steadily decreasing. In 1885 it amounted to £735 15*s.* 9*d.*; in 1886, to £691 16*s.* 8*d.*; and in 1877 to £614 1*s.* 9*d.*! Mr. R. M. Craven, the chairman of the Hospital Sunday Committee, in alluding to this decrease at a meeting of that body, attributed it to the recent hard times, and looked hopefully forward to a speedy move in the opposite direction as soon as trade should revive. Alderman Witty, who seconded the chairman's remarks, said he looked confidently forward to the collections increasing to £1,000 in future years. But even this ultimatum seems to us but a small sum for a busy, industrial town to contribute to such an important object.

ACCORDING to the annual report of the Tunbridge Wells and Rusthall Provident Dispensary, that institution seems to be well worked, and to give satisfaction both to subscribers, patients, and doctors. The persons entitled to benefits during the past year amounted to 2,635, and the number now on the books is 2,105, of whom 1,325 are above fourteen years of age and the rest are children. It is stated that more than 100 cases are constantly on the lists of the visiting medical men, and that the number of prescriptions sent in for dispensing during the past year has been 29,567. The sum left for division amongst the medical officers under Rule III. is £400 13*s.* 7*d.* Considering the enormous amount of work represented by the number of patients on the lists of the medical men, and the extraordinary total of nearly 30,000 prescriptions to be dispensed, it is difficult to see now such a comparatively small sum could give anything like adequate remuneration to the medical men; of that, however, they must be considered to be the best judges, and if they are pleased, nobody else has any right to be dissatisfied.

THE following vacancies are announced this week, the amount of salary in each case being given after the name of the institution :—

**Resident Medical Officers.**

Hull Royal Infirmary Junior Assistant House Surgeon.—£50.
Monsall Fever Hospital, Manchester. Assistant Medical Officer. £100.
Hospital for Sick Children, Great Ormond Street, W.C. Medical Officer, as House Surgeon.—£50.
Royal Free Hospital, Gray's Inn Road, W.C. Junior Medical Officer.
Royal Hospital for Diseases of the Chest, City Road. Senior House Physician.—£80.
Sussex County Lunatic Asylum, Hayward's Heath. Medical Superintendent.—Salary £600, with furnished house, coal, gas, vegetables, milk and washing.
Sussex County Lunatic Asylum, Hayward's Heath. Junior Assistant Medical Officer.—£100, with board and washing.

**Non-resident Medical Officers.**

Poplar Hospital for Accidents, East India Road, E.C. Two Honorary Surgeons.
Essex and Colchester Hospital. Physician.—£200.
St. John's Hospital for Diseases of the Skin. Two Assistant Physicians.

**Matron.**

Cumberland Infirmary. Matron wanted.—£60, with board, lodging, and washing.

**Nurses.**

Portsmouth Royal Hospital. Trained Nurse for Male Surgical Wards.—£25, with uniform
Kingston House, Baker Street, Hull. Thoroughly trained Nurses wanted.
Cottage Hospital, Eltham, Kent. Trained Nurse.
Workhouse Infirmary Nursing Association Trained Nurses, to receive training as Midwives
Belgravia Institute for Trained Nurses. Medical and Surgical Nurses.

---

THE total bequests and donations to the Belfast Royal Hospital during the year amount to £5,723 9*s*. 5*d*., showing an increase over last year of £72 18*s*. In spite of this, however, the hospital still suffers from a deficit of £581 8*s*. 1*d*.

---

MR. T. B. HENDERSON writes to the *Heckmondwike Herald*, suggesting another method of taking up the subscriptions now collected *en masse* on Hospital Saturday. These are mostly obtained in sixpences and shillings on a special day, and he points out that it would be easier for working men to part with a small sum, from a halfpenny upward, every week, than to give a larger sum on a single occasion; aad they would thus greatly enlarge their subscriptions without feeling any strain. This plan, it will be remembered, was suggested by the Duke of Argyll in his recent article in THE HOSPITAL, and his Grace then gave statistics showing how admirably successful it had been in Glasgow.

---

THE Exeter Hospital Saturday Fund amounted last year to £477 4*s*. 5*d*., a decrease of about £21 compared with the preceding year. The ex-mayor (Mr. Burch), who presided at the committee meeting where the report giving these figures was read, stated that he considered the diminution to be due to the considerable demands made upon the liberality of the inhabitants in connexion with the theatre disaster; and he was glad to say that the Fire Relief Committee had very liberally recouped to the hospital all the expenses they had incurred in connexion with the sufferers taken there on the occasion of the fire.

---

THE annual report of the East London Hospital for Children and Dispensary for Women, situated at Shadwell, states that the total number of cases treated there last year amounts to 16,000 cases. The admission to the in-patients' wards is restricted to children, but women are treated at the dispensary as out-patients. Of the 1,000 children received in the hospital during the year, 260 died in the wards. Of these 180 were infants under two years of age, and 41 died a few hours after admission. This high rate of mortality shows the effect on health and life of that neglect and insufficiency of food from which the children of the poor so often suffer.

ALL who desire to see nurses receive the social recognition their noble and unselfish labours deserve, will be touched and gratified by the act by which M. Sadi Carnot, the recently-elected President of the French Republic, signalised the last day of the departing year. We mean the bestowal of decorations on Mdlle. Nicole, who for thirty-six years has devoted herself to arousing and developing the dormant faculties of the idiots in the Salpêtrière, and the Sister of Charity in charge of the military hospitals of Val de Grace. The spontaneous and unpremeditated manner in which this graceful act was done adds to its value, as proving that it was the outcome of personal respect for the two nurses, and admiration for work as remarkable as it was praiseworthy. M. Carnot, whose creed is humanitarian in the highest sense, was paying a visit to the hospitals of Paris when he saw these ladies, and learned for the first time all they had done. Struck with surprise, and anxious to show his appreciation of such devoted efforts, he borrowed from the head physicians of the respective establishments, who happened to be knights, their ribbons of knighthood, and gave them to the nurses. Mademoiselle Nicole, who was first admitted to the Salpêtrière to attend her mother, who had gone mad, and since her death has devoted herself to the idiot children in the hospital, fainted with agitation when the honour was conferred on her.

---

ON Wednesday, the 28th ult., Holy Innocents' Day, a special service for children was held in Westminster Abbey. The Dean in his sermon referred to the children interred within the Abbey—the Princess Mary, a two-year old namesake of the Queen of Scots, and the first child born to James I. after he ascended the Throne of England; and the young uncrowned Edward V. and his brother, murdered in the Tower. At the end of his sermon Dr. Bradley asked his hearers for contributions to the Destitute Childrens' Dinner Society.

---

THE small-pox epidemic at Sheffield has been steadily increasing up to the present time. It began in March, during which month there were three cases; in April, 4; May, 21; June, 43; July, 91; August, 146; September, 275; October, 498; November, 604; and up to December 19th, 585. Dr. Sinclair White, the medical officer of health, complains of the difficulty experienced in hearing of the existence of fresh cases, and points to Leicester, where the authorities have power under a local act to compel notification of infectious diseases, thereby facilitating the stamping out of the epidemic. The contractor for the preparation of the new small-pox hospital has undertaken to complete it by January 15th, and with its aid it is to be hoped that the prompt and complete isolation of patients will help to stamp out the epidemic, though in the face of present statistics its provision of 100 beds seems somewhat inadequate.

---

THE hospital authorities at Leeds cannot but feel gratified at the testimony to the value and efficiency of the institution, shown in the voluntary gift to it of two ambulances by the working men and women of the district. The larger ambulance cost £158, and the smaller £90. The total amount collected in 148 workshops and 76 trade and friendly societies was £328 18*s*. 3*d*.; and the largest subscription came from the work-girls at Messrs. John Barran and Sons' factory. It is believed that after paying other expenses there will be a sufficient balance left to permit of the committee connecting the hospital with the fire brigade station, where the ambulances are to be kept. The firemen, it is said, are very proud of having the care of the ambulances committed to their care. There is no more satisfactory sign of the times than the increasing desire shown by the working classes to help in the support of institutions from which they profit so much; and we especially congratulate the work-girls of Leeds for the large share they have subscribed for this valuable and useful gift.

